# Clinicopathological Profile of Breast Cancer at a Tertiary Cancer Center in Jharkhand, India: A Descriptive Cohort Study

**DOI:** 10.7759/cureus.39990

**Published:** 2023-06-05

**Authors:** Amitabh Kumar Upadhyay, Aaditya Prakash

**Affiliations:** 1 Medical Oncology, Tata Main Hospital, Jamshedpur, IND; 2 Radiation Oncology, Tata Main Hospital, Jamshedpur, IND

**Keywords:** clinicopathological profile, profile, india, jharkhand, breast cancer

## Abstract

Introduction

Breast cancer is the most common cancer in females worldwide including Indian urban areas. There is no concrete data on breast cancer epidemiology from the state of Jharkhand, India.

Materials and methods

The present study is a retrospectively conducted descriptive cohort study. A total of 759 patients were selected from the database from 2012 to 2022. The parameters taken for the study were age, sex, stage at the time of presentation, histological type, estrogen receptor (ER) status, progesterone receptor (PR) status, human epidermal growth receptor 2 (HER2) neu status (HER2/neu), site of metastasis for stage 4 diseases, parity, and significant family history.

Results

The median age for patients was 49 years (range: 19-91 years), with a clustering of 74.83% of cases between 31 and 60 years of age. Most of the patients were in stage III, with 365 (48.08%) cases. Bone was the commonest site of metastasis and was found in 41.25% of total cases. The total number of hormone receptor-positive patients was 384 (56.2%), the number of HER2/neu positive patients was 210 (30.7%), and triple-negative breast cancer was found in 184 cases (26.93%).

Conclusion

The pattern found in our Jharkhand patients was very much similar to other Indian studies with slightly more clustering of younger cases. The cases in India are almost a decade younger than the Western population and the same was replicated in our study. This is one of the largest studies on breast cancer profile and epidemiology from the eastern part of India. Most of our patients presented late, leading to a higher number of locally advanced (stage III) and metastatic (stage IV) cases. More awareness is required at the population level, including strict implementation of a robust screening program by our government, for improving the overall outcome.

## Introduction

Carcinoma of the breast is the most common cancer in females worldwide and has now surpassed carcinoma of the lung as the leading cause of cancer in the year 2020, with an estimated 2.3 million new cases, representing 11.7% of all cancers [1]. Epidemiological studies have projected a persistent rising trend with almost 3 million new cases of breast cancer by 2040 and 1 million deaths every year because of population growth and aging alone [2]. In India, it is the commonest cancer among females in urban areas, accounting for approximately 30% of all cancers, while cervical cancer continues to be rampant in most rural areas [3-5]. The incidence in India has been increasing significantly in the last 50 years, and between 1990 and 2016, the age-standardized incidence rate of carcinoma breast has increased by 39.1%, with a similar trend reported in every region of the country [3-5]. As per the Indian Council of Medical Research (ICMR) statistics, the crude rate for breast cancer was between 4.5 and 39.0 and age-adjusted risk was between 7.0 and 48.0 per one lac population across various regions of India [3]. Breast carcinoma accounted for 13.5% of all cancers and 10.6% of all cancer deaths in India as per the Globocan Data 2020 [6].

Breast cancer is a heterogeneous disease with many factors influencing its prognosis. The factors that affect its prognosis are age, sex, stage, histological type, grade, hormonal status, human epidermal growth receptor 2 (HER2)/neu status, and received treatment, among others. There are many large-scale studies on the clinicopathological profile of breast cancer from different parts of India, but there are no concrete data on breast cancer epidemiology from the state of Jharkhand, India. Therefore, this study on the clinicopathological profile of breast cancer was planned at Tata Main Hospital (TMH) and Meherbai Tata Memorial Hospital (MTMH), Jamshedpur, which is the largest tertiary cancer center in the state of Jharkhand.

## Materials and methods

The present study is a descriptive cohort study that was conducted retrospectively after taking administrative approvals from TMH and MTMH. Institutional ethics committee approval was not taken since its retrospective data analysis. Both these hospitals cover urban areas of Jamshedpur as well as most of the rural areas of many Jharkhand districts and some districts of neighboring states such as West Bengal and Odisha. A total of 759 patients with breast cancer were selected from the database from January 2012 to December 2022. The minimum criteria for inclusion were the availability of information regarding clinical or pathological stage and histological type on biopsy or post-operative histopathology along with basic parameters such as age and sex. Patients with incomplete documentation of stage and histological type were excluded. The parameters taken for the study were age, sex, stage at the time of presentation, histological type, estrogen receptor (ER) status, progesterone receptor (PR) status, HER2/neu status, site of metastasis for stage 4 diseases, parity, and significant family history. Mammography was performed in all cases with the exception of ulcerated breast cancers, where ultrasound breast and axilla were performed for local imaging. Technetium 99m methylene diphosphonate (99mTc-MDP) bone scan was performed in locally advanced, metastatic cases and clinically early breast cancer cases with raised alkaline phosphatase levels in liver function tests or having symptoms of bony metastasis. Contrast-enhanced computerized tomography (CECT) or whole body positron emission tomography (PET) scan was performed for metastatic workup of eligible cases. MRI of the brain, plain plus contrast, was performed in selected cases with signs of brain metastasis or suspicious lesion found in PET scan or CECT scans. The immunohistochemistry (IHC) method was used to determine ER, PR, and HER2/neu status on either pre-operative biopsy or post-operative tissue using the standard method of sectioning paraffin-embedded tissue and staining with monoclonal antibodies for ER, PR, and HER2/neu. Nuclear staining of more than 1% was definition for ER and PR positivity. Patients with HER2/neu score 3+ or fluorescence in situ hybridization (FISH) positive (more than six copies of HER2/neu gene or HER2/CEP17 ratio of more than 2) were defined as HER2 positive by the DAKO Hercept test. All patients with HER2/neu scores of 2+ were subjected to the FISH method for confirming HER2/neu amplification. The patients with any first- or second-degree family member with a history of related cancers such as carcinoma ovary/ fallopian tube were labeled as significant family history.

Statistical analysis

Continuous variables were expressed as mean (SD) and ranges, and categorical variables were expressed as numbers and percentages. Comparison between two categorical variables was assessed using the chi-square test. All tests were two-sided. A p-value of <0.05 was significant. The analysis was conducted using SPSS Version 21.0 (IBM Corp., Armonk, NY).

## Results

A total of 759 patients were included in the study after excluding 35 cases in which minimum requirements were not fulfilled. Most of the patients were 31-60 years of age (74.83%). The median age for patients was 49 years (range: 19-91 years), and the average age was 50.3 years (SD: 10.4). Out of 575 patients, 11 were male, accounting for 1.4% of cases. There were four patients with synchronous bilateral breast cancer (Table 1).

**Table 1:** Clinical profile of cases

Name of variables (n)	Categories	n (%)
Sex (n=759)	Male	11 (1.45)
	Female	748 (98.55)
Age (n=759)	<20 years	1 (0.3)
	21-30 years	31 (4.08)
	31-40 years	140 (18.44)
	41-50 years	240 (31.62)
	51-60 years	188 (24.76)
	61-70 years	108 (14.22)
	71-80 years	43 (5.66)
	≥80 years	8 (1.05)
Stage (n=759)	0	4 (0.52)
	I	22 (2.89)
	II	218 (28.72)
	III	365 (48.08)
	IV	160 (21.08)
Histology (n=759)	Ductal	698 (91.96)
	Lobular	13 (1.71)
	Medullary	9 (1.85)
	Tabular	3 (0.39)
	Mucinous	12 (1.58)
	Papillary	10 (1.31)
	Neuroendocrine features	1 (0.3)
	Metaplastic carcinoma	8 (1.05)
	Phyllodes tumor	5 (0.65)
Parity (n=557)	Nulliparous	32 (5.74)
	Uniparous	62 (11.13)
	Multiparous	463 (83.12)

Lump in the breast was a major complaint in 74% of patients, followed by pain in the breast in 12% of cases, ulceration in 6% of cases, nipple discharge in 3% of cases, and complaint not related to the breast in 5% of cases.

Most of the patients were in stage III, with 365 (48.08%) cases, followed by stage II in 218 (28.72%) cases, stage IV in 160 (21.08%) cases, and stage 1 in 22 (2.89%) cases. DCIS (stage 0) was found in four (0.52%) patients only (Table 1).

The most common histological type as per pre-operative biopsy/post-operative histopathology was ductal carcinoma in 698 (91.96%), which is the most common type as per literature [3-8]. We also found rare types such as lobular in 13 patients, mucinous in 12 patients, medullary in nine patients, papillary in 10 patients, tubular in three patients, and neuroendocrine carcinoma in one case. Metaplastic carcinoma was found in eight cases, and phyllodes tumor was found in five cases (Table 1).

Data on parity could be found in 557 patients out of 759 total sample size. Nulliparity was found in 5.74% of cases, uniparous patients were found in 11.13% of cases, and multiparous patients were found in 83.12% of cases (Table 1). A positive family history was found in 23 cases, accounting for 7% of cases.

Out of 160 stage IV cases, the metastatic pattern is shown in Table 2. Bony metastasis was the commonest presentation in the study population.

**Table 2:** Site of metastasis in stage IV patients

Site of metastasis in stage IV patients (n=145)	n (%)
Bone	66 (41.25)
Lung	51 (31.87)
Liver	29 (18.15)
Pleural	15 (9.37)
Brain	10 (6.25)
Adrenal	3 (1.87)
Non-regional lymph nodes	27 (16.87)

The data on ER, PR, and HER2/neu status were found for 683 patients only out of a total sample size of 759 patients due to a lack of adequate documentation in older records. The total number of hormone receptor-positive patients was 384, accounting for 56.2% of 683 cases. The total number of Her2/neu-positive patients found by either IHC or FISH was 210, accounting for 30.7% of cases. Triple-negative breast cancer (TNBC) was found in 184 cases, accounting for 26.93% of cases (Table 3).

**Table 3:** Distribution of patients as per receptor status ER, estrogen receptor; PR, progesterone receptor; HER2, human epidermal growth receptor 2; IHC, immunohistochemistry; FISH, fluorescence in situ hybridization; TNBC, triple-negative breast cancer

Category (n=683)	n (%)
Hormone receptor-positive (ER- and/or PR-positive)	384 (56.22)
HER2 positive (by IHC or FISH)	210 (30.74)
Hormone receptor-positive plus HER2 positive	95 (13.90)
Hormone receptor-positive plus HER2 negative	289 (42.31)
Hormone receptor-negative plus HER2 positive	115 (16.83)
Triple-negative (ER/PR/HER2 negative, TNBC)	184 (26.93)

Out of non-metastatic and metastatic breast cancers (MBCs), the distribution of HR-positive disease was different with more HR-positive cases (57.43%) in non-metastatic cases versus metastatic cases (51.72%). The HER2/neu positivity increased from 29.55% in non-metastatic to 35.17% in MBC. TNBC rates increased from 25.46% in non-MBC to 32.45% in MBC. We could not find any significant difference (p = 0.190), though there is a numerical difference probably due to a smaller sample size (Table 4).

**Table 4:** Characterization of patients among non-metastatic and metastatic diseases MBC, metastatic breast cancer; HR, hormone receptor; HER2, human epidermal growth receptor 2; TNBC, triple-negative breast cancer

	Stage (n=683)		P-value
	Non-MBC (n=538)	MBC (n=145)	
HR+	309 (57.43)	75 (51.72)	0.190
HER2/neu positive	159 (29.55)	51 (35.17)	
TNBC	137 (25.46)	47 (32.45)	

## Discussion

Breast cancer is the most common cancer in females overall, but there are a lot of variations among different socioeconomic profiles of patients across different geographical areas. The incidence of breast cancer is steadily increasing due to lifestyle, changes and increasing age is one of the most important risk factors. A high-calorie diet along with a lack of physical activity, altered reproductive pattern, early menarche and late menopause, and decreased parity are other risk factors for breast cancer. The median age for breast cancer in India is 47-50 years, which is almost a decade younger than that in the Western population, where the median age is around 60 years, and the same was replicated in our study [1-21]. The distribution of patients above 50 years and below 50 years is 46% and 54%, respectively, in the current study. The age group of 31-50 years accounted for 50.1% of cases, and the age group of 31-60 years accounted for 74.83% of cases. According to a report of Hospital-Based Cancer Registries (2007-2011), 65-70% of patients were older than 50, and 30-35% of patients were younger than 50 years [11]. The incidence is rising in India, with a peak at 50-60 years, according to the National Cancer Registry Program [3-5]. Cases younger than 50 years accounted for 65.8% of the total. The pattern found in our Jharkhand patients was very much similar, with slightly more clustering of younger cases with a median age of 49 years and an average age of 50.3 years. Lump in the breast with associated pain, discharge, and ulceration was the most common presenting symptom, and this is the usual pattern of presentation worldwide [7-21].

The stage of presentation in Indian patients is very different from the Western cohort. Most of the patients in the Western cohort report in early stages, while cases with locally advanced breast cancer (LABC) and metastatic cancers are very less. In a study by Nada Alwan et al., the quantum of LABC was 2.4%; in addition, stage IV was found in 0.7%, stage I was found in 59.1%, and stage II was found in 37.8% in the British population [7]. According to the North American Association of Central Cancer Registries (NAACCR) 2019, breast cancer in the USA during 2012-2016 had a similar presentation, with stage I accounting for 47%, stage II for 43%, stage III for 7%, and stage IV for 3% cases at the time of presentation [8]. We found stage III in 48.08%, stage IV in 21.08% stage I in 2.89%, and stage II in 28.72% of cases. Most of the Indian studies have shown similar results, showing a higher number of cases with advanced stage of presentation [12-15]. The probable reason for this advanced presentation is multifactorial, such as low socioeconomic status, lack of awareness, lack of robust screening program, lower education level, lack of adequate healthcare resources, and considering cancer as a social stigma, hence leading to social embarrassment and isolation. The majority of rural patients ignore the lump or symptoms for a very long time. The lump is usually asymptomatic, which leads to delay in seeking medical advice in more than 50% of the cases in rural areas [3]. Most of the cases report to the hospitals only when there is a huge lump or secondary changes such as local skin ulcerations are obvious. Non-availability or scarcity of specialized cancer treatment centers nearby is also a major factor in not being able to seek early treatment. Patients are afraid of cancer morbidity and mortality and also have apprehension that their family’s reputation will be affected if the news is broken in society, including potential difficulties in their daughter’s marriage also. This late presentation leads to poorer outcomes as compared to Western counterparts. The majority of our rural patients are very poor and cannot afford the complete treatment cost. Usually, there is a single bread earner in the family and that daily income also gets affected while getting the workup and treatment. Most of our cases take support from the government scheme or the Ayushman Bharat Pradhan Mantri Jan Arogya Yojana (PM-JAY) scheme for treatment. Screening is very important for early diagnosis, and there are many factors that make early diagnosis or screening difficult for rural populations, such as non-availability of such diagnostic facilities in the nearby area, patient’s willingness, and the time required for traveling to the diagnostic centers leading to daily wage loss. Very few women perform a self-breast examination, opt for a routine clinical examination by a doctor, or undergo mammography for screening. Gradually, many screening centers are opening up in the country. Slowly, this situation is improving with time due to various awareness initiatives, improved education levels, and better internet access and usage. With the start of PM-JAY, almost 50% of the population can potentially be covered for cancer treatment under this national health insurance cover. The likely impact of this policy on overall epidemiological distribution and outcome will take many years to match the distribution in Western countries.

The histological distribution of cases was similar to other Indian studies [14-21] (Table 5).

**Table 5:** Comparison of our study with large-scale studies from India EBC, early breast cancer; LABC, locally advanced breast cancer; ER, estrogen receptor; PR, progesterone receptor

Authors	Study period	Number of cases	Median age	Hormone receptor-positive (%)	HER2/neu positive (%)	Triple negative (%)
Ghosh et al. [17]	2008	2001	49	51	16.7	29.8
Doval et al. [18]	2008-2011	1284	52	63	23	23.8
Gogia et al. [14]	2014-2016	550	48	59	29	28
Nair et al. [15]	2009	709 EBC	50	58.7	17	28.5
		688 LABC	48	51	15.6	36.1
Raina et al. [19]	1993-1999	173 EBC	47	53.7	NA	NA
Kumar et al. [20]	2010- 2016	5,436	48	48	15	37
Datkhile et al. [21]	2014-2019	350	50	ER: 55.1; PR: 51.4	14.57	33.14
Current study	2012-2022	683	49	56.2	30.7	26.93

Out of 145 stage IV cases, bone metastasis was the most common site in 40.9% of cases, followed by lungs in 31.9%, liver in 18%, non-regional lymph nodes in 18%, pleural in 8.2%, brain in 3.2%, and adrenal in 0.8% cases. In a study by Gogia et al. [14], bone metastasis was found in 48.5%, followed by lungs in 38.3%, liver in 26.2%, non-regional lymph nodes in 15.1%, and brain in 5%. The pattern of metastasis is very much similar in our patients. The pattern of male breast cancer was similar, with the average age being 49.5 years in our 11 cases out of 759 patients, accounting for 1.44% of the total [14-22].

There is a lot of heterogeneity in hormone receptor and HER2 positivity rates in our country. Patients with hormone receptor positivity were 56.22%, those with HER2/neu positivity were 30.74%, and triple-negative cases were 26.93%. Hormone receptor positivity has varied widely in various Indian studies between 48% and 63% (Table 5). In a large-scale study from Kerala, India, ER positivity was found to be 52% [23]. The average hormone receptor positivity for white women in the USA was found to be 77% in another study [10].

HER2/neu positivity was present in 30.7% of our cases, and in various large-scale studies from India, it has varied between 14.7% and 29% (Table 5). In Western studies, HER2 positivity has been reported to be from 17% to 27% [24,25]. A study from Malaysia showed HER2 positivity of 31.5% in breast cancers [26]. HER2 positivity was seen in 11.4% of cases in Japan as per the annual report of the National Clinical Database in 2018 [27]. The HER2/neu positivity rate has been variable among some other Indian studies also. A study from Bengaluru, South India, found 43.2% HER2 positivity by IHC [28]. A study from Indore, Central India, has shown 40.2% HER2 positivity [29], and a study from Varanasi, North India, has shown 46.3% HER2 positivity [30].

HER2/neu positivity confers a prognostic disadvantage over other subtypes. The prognosis can be improved with anti-HER2 drugs such as trastuzumab, pertuzumab, trastuzumab emtansine (TDM1), lapatinib, neratinib, tucatinib, and fam-trastuzumab deruxtecan, but affordability is a big concern in this part of the country despite the availability of generics for trastuzumab and TDM1. More than 60% of patients do not take any anti-HER2 therapy despite having HER2-positive status in tumors at our hospital. Though our study is covering only the clinical profile, affordability for anti-HER2 therapies is a major bottleneck for the optimum treatment of our cases. More than 25% of cases do not complete the planned treatment due to poor financial status. Overall, these factors have a significant contribution to the poor outcome of our cases. The overall proportion is numerically different as compared to other large Indian studies with hormone receptor-positive cases within range and comparatively a greater number of patients with HER2/neu positive status (Table 5). In a large country like India, some variations are seen and very much expected among various states and geographical areas, probably due to different demographic profiles, genetics, and social status.

Limitations of study

The treatment part and survival analysis were not included in this study since this was not part of the protocol. Data on ER, PR, and HER2/neu were not available for 76 out of the total sample size of 759 patients.

## Conclusions

This is one of the largest studies on breast cancer profile and epidemiology from the eastern part of India. There is no such publication from the state of Jharkhand to the best of our knowledge. Since the study covers most of the rural population, it can be concluded that this study reflects the current situation in rural areas of Jharkhand. Most of our patients present late, leading to a greater number of locally advanced tumors and a very high number of stage IV cases. We also have a younger age of presentation and a lesser number of hormone receptor-positive patients compared to the Western counterparts, leading to poorer outcomes as well. More awareness is required at the population level, including strict implementation of screening programs by our government for diagnosis at an earlier stage.
